# Supplementary material for: The Effect of Staging Intervals on Progression-Free Survival in Registration Studies of Oncologic Drugs: A Meta-Analysis
Source: Cancers (Basel). 2025 Apr 18;17(8):1359. doi: 10.3390/cancers17081359 (PMC12025954; doi:10.3390/cancers17081359)
Supplement: Supplementary file 1 [file cancers-17-01359-s001.zip › Supplementary Table S1.pdf]

Staging Interval (weeks)	HR for PFS (95% Cl)	*P* for Subgroup Difference	Heterogeneity I^2
**Intervals**			
< 8 (n=48) vs. ≥ 8 (n=57)	0.58 (0.52, 0.64) vs. 0.49 (0.44, 0.53)	0.01	90%
< 9 (n=85) vs. ≥ 9 (n=20)	0.54 (0.50, 0.58) vs. 0.47 (0.40, 0.56)	0.18	90%
< 12 (n= 91) vs. ≥ 12 (n= 14)	0.55 (0.51, 0.59) vs. 0.39 (0.33, 0.46)	<0.001	90%
< 8 (n= 45) vs. 8 ≥ (n= 53) without < 6 and > 12	0.60 (0.54, 0.66) vs. 0.50 (0.45, 0.54)	0.005	89%
≤ 6 (n= 48) vs. > 6 to < 12 (n= 43) vs. ≥ 12 (n= 14)	0.58 (0.52, 0.64) vs. 0.52 (0.47, 0.58) vs. 0.39 (0.33, 0.46)	<0.001	90%
**Drug Class (staging interval < 8 weeks vs. ≥ 8 weeks)**			
Immunotherapy (n=25 vs. n=11)	0.70 (0.63, 0.78) vs. 0.66 (0.56, 0.78)	0.51	84%
Small Molecules (n=16 vs. n=31)	0.42 (0.36, 0.49) vs. 0.47 (0.42, 0.52)	0.3	81%
Antibodies (n=6 vs. n=3)	0.54 (0.41, 0.70) vs. 0.55 (0.43, 0.70)	0.88	89%
**Indication group (staging interval < 8 weeks vs. ≥ 8 weeks)**			
Breast (n=5 vs n=12)	0.49 (0.36, 0.66) vs. 0.58 (0.52, 0.65)	0.31	76%
Lung (n=15 vs. n=7)	0.60 (0.51, 0.69) vs. 0.60 (0.46, 0.77)	0.98	88%
GI (incl. HCC) (n=7 vs. n=6)	0.57 (0.48, 0.68) vs. 0.53 (0.46, 0.61)	0.52	79%
Melanoma (n=7 vs. n=5)	0.44 (0.34, 0.57) vs. 0.60 (0.52, 0.70)	0.04	80%
Kidney (n=6 vs. n=2)	0.67 (0.55, 0.81) vs. 0.44 (0.34, 0.58)	0.01	89%
Sarcoma and GIST (n=3 vs. n=1)	0.33 (0.11, 0.98) vs. 0.31 (0.24, 0.40)	0.98	96%

Supplementary Table S1. Sensitivity analyses for Phase 3 studies only
